# Numerical Analysis of Mixing Performance in an Electroosmotic Micromixer with Cosine Channel Walls

**DOI:** 10.3390/mi13111933

**Published:** 2022-11-09

**Authors:** Zhong Chen, Yalin Wang, Song Zhou

**Affiliations:** Jiangsu Key Laboratory of Advanced Manufacturing Technology, Huaiyin Institute of Technology, Huaian 223003, China

**Keywords:** numerical simulation, micromixer, electroosmotic, mixing performance

## Abstract

Micromixers have significant potential in the field of chemical synthesis and biological pharmaceuticals, etc. In this study, the design and numerical simulations of a passive micromixer and a novel active electroosmotic micromixer by assembling electrode pairs were both presented with a cosine channel wall. The finite element method (FEM) coupled with Multiphysics modeling was used. To propose an efficient micromixer structure, firstly, different geometrical parameters such as amplitude-to-wavelength ratio (a/c) and mixing units (N) in the steady state without an electric field were investigated. This paper aims to seek a high-quality mixing solution. Therefore, based on the optimization of the above parameters of the passive micromixer, a new type of electroosmotic micromixer with an AC electric field was proposed. The results show that the vortices generated by electroosmosis can effectively induce fluid mixing. The effects of key parameters such as the Reynolds number, the number of electrode pairs, phase shift, voltage, and electrode frequency on the mixing performance were specifically discussed through numerical analysis. The mixing efficiency of the electroosmotic micromixer is quantitatively analyzed, which can be achieved at 96%. The proposed micromixer has a simple structure that can obtain a fast response and high mixing index.

## 1. Introduction

Micromixers, such as lap-on-a-chip systems, can be regarded as one of the most important devices in micromachines and have played an essential role in a wide variety of applications, such as biomedical diagnosis, chemical detection, and drug delivery [1]. This makes it an important and challenging operation to realize efficient and rapid mixing of two or more fluids. The miniaturization characteristics restrict turbulence from occurring with low Reynolds numbers. The mixing behavior is primarily dominated by molecular diffusion, namely laminar flow [2]. The slow response process and long mixing channels limit the effective mixing of samples in the detection operation and increase the experiment cost. Therefore, with the micrometer scale characteristics of microfluidic devices, improving the mixing quality of micromixers even at relatively low Reynolds numbers is a subject that we need to continue to pay attention to and study.

To enhance the mixing performance, researchers have developed many micro-mixers, which easily achieve mixing, separation, and chemical reactions and can be roughly divided into passive and active mixers. Most studies have shown that by changing the complex structural design in passive micromixers, fluids can be stretched, deformed, and flowed along the wall to achieve effective mixing [3]. Passive micromixers have the advantages of ease of manufacture and simplicity over active mixers, but molecules mix slowly in this case, so a rapidly responsive mixing system must be found instead. Also, it is very important to understand the mixing process inside the mixer to improve the mixing performance. Therefore, through qualitative and quantitative analysis of flow mechanisms and mixing performance, many effective methods have been developed to enhance the fluids mixing in passive micromixers, such as laminar flow micromixers [4,5], chaotic convection [6,7,8], split-recombination [9,10] and split-confluence [11,12] micromixers. The mixing of fluid samples is achieved by generating secondary flow and reflux phenomena. Researchers have recently discovered that micromixers with trigonometric wall channels and convergent-divergent geometries have been numerically and experimentally demonstrated to significantly affect fluid mixing [13,14]. With respect to active micromixers, fluid mixing is realized by perturbing the fluid with the help of additional components or energy sources, such as acoustics [15], magnetic fields [16], and electricity [17]. The electroosmotic flow (EOF) has attracted more attention in developing the perfect mixing performance of microfluidic systems. Besides, the fluid flow and mass transport can be numerically simulated by FEM coupled with Multiphysics software [18].

Previous studies have indicated that there are different designs to improve the mixing quality based on the EOF method for Newtonian and non-Newtonian fluids. For one design, electrokinetic flows were performed by using a regularly switching electric field to induce electroosmotic flows. [19,20]. Another method is to apply non-uniform zeta potential on inlets and outlets to generate vortices on the charged surface. In terms of the above convergent-divergent geometries of microchannels, several studies were concerned with developing EOF mechanisms on wavy microchannel surfaces. Ching-Chang Cho et al. [21] numerically investigated the flow behavior of electrokinetically-driven non-Newtonian fluids in a microchannel. This study especially emphasized the microchannel roughness of a complex-wavy surface. The study revealed that the flow behavior index using a power-law model has an influence on the flow field characteristics. A. Banerjee and A.K. Nayak [22] carried out simulations of electrokinetically driven flow in a wavy-structure to study the mixing index and pressure drop by changing zeta potentials. In this research, an improvement in pressure gradient can be observed for the wavy microchannel compared to the straight one. Sumit Kumar Mehta et al. [23] conducted numerical simulations of the non-Newtonian fluids mixing by vortex-induced electroosmosis in a wavy micromixer. They have considered the effect of the nonuniform surface potential and the phase lag between surface potential. They concluded that moderate modulation of phase lag and lower surface potential could bring about efficient mixing. Recently, Sumit Kumar Mehta and Sukumar Pati [24] investigated the effect of phase lag (Δϕ) between the wavy walls by utilizing surface charge heterogeneity for different values of the diffusive Peclet number (Pe), Debye parameter (κ), geometrical wave number (n), and dimensionless wall amplitude (α). The results showed that the phase lag of surface charge heterogeneity could take stronger action on the mixing performance with the above other parameters. They also found that for thinner EDL (κ = 150), the mixing index can achieve larger than 90% up to higher values of Pe with a higher flow rate at Δϕ = π/2 and π. It is worth noting that great progress has been made in enhancing the efficiency of fluid flow through the wavy channel in the above investigations.

Alternating current electroosmotic (ACEO) micromixers are widely used because of their easy integration and high reliability. In this type of micromixer device, the use of electroosmotic flow to stir the fluid is a popular method. The input signal acts on the surface of the microelectrode, which can generate induced charge so that electrical double layer (EDL) is performed. The charge in the diffusion layer in the EDL moves directionally under the action of the tangential electric field to form an electroosmotic flow. It is worth noting that the above phenomenon is affected by micromixer structures and depends on the frequency, voltage, and other characteristics of the AC electric field. Mirzakhanloo et al. [25] presented a T-shape electroosmotic micromixer with a chamber in the middle. The influence of several geometry parameters on mixing quality was simulated by numerical analysis. This analysis revealed the meaningful influence of micromixer design on the mixing quality. Amir Shamloo et al. [26] pointed out two-dimensional micromixer models with AC electroosmosis through three different geometries: one-ring, diamond, and two-ring. It should be mentioned that employing the one-ring geometry under the most favorable conditions combined with pi radian phase lag can achieve a high mixing performance (99.4%). They also found that the effective mixing in micromixers is made available for both Newtonian and non-Newtonian fluids in terms of blood for biological applications. Cao et al. [27] used 16 pairs of electrodes embedded in the microchannel with an optimum location to enhance the mixing quality. Usefian et al. [28] presented a novel type of electroosmotic micromixer under AC and DC electric fields. PDMS and gold nanoparticles were employed for making microchips and electrodes, respectively. As the results show, in both cases, the mixing performance can be enhanced by altering the voltage value and the fluid inlet velocity. The simulated results are in qualitative agreement with experimental results. Most recently, Cheng et al. [29] compared three forms of voltage functions applied on the electrodes of channel walls for the mixing performance of a T-shaped micromixer. They found that the mixing efficiency first increases and then decreases with the increase of the frequency values in the wide range of 50 Hz to 400 Hz. Moreover, the best mixing for the three cases can be achieved at 200 Hz.

Several works considered the influence of wavy channels and electric fields to investigate the flow properties in the micromixers. Accordingly, there is little attention on embedding the electrode pairs on the wavy channel walls. In this present study, a simple passive micromixer was presented with a cosine microchannel wall, as well as a novel ACEO micromixer equipped with two pairs of electrodes. To deeply investigate the mixing performance of the proposed micromixer, the structural design of the passive micromixer in a steady state and the electrode parameters of the ACEO micromixer are considered in a transient state by means of a sinusoidal AC electric field. By performing Multiphysics numerical simulations, the introduced AC electric field can cause intense motion, which was compared with the passive one. The high-quality mixing is finally achieved under the condition that the fluid flow at the inlet of the microchannel is guaranteed to be stable. The novel micromixer can provide a new idea for fluid mixing and subsequent applications, such as chemical reactions, etc.

## 2. Micromixer Design

**Figure 1:**
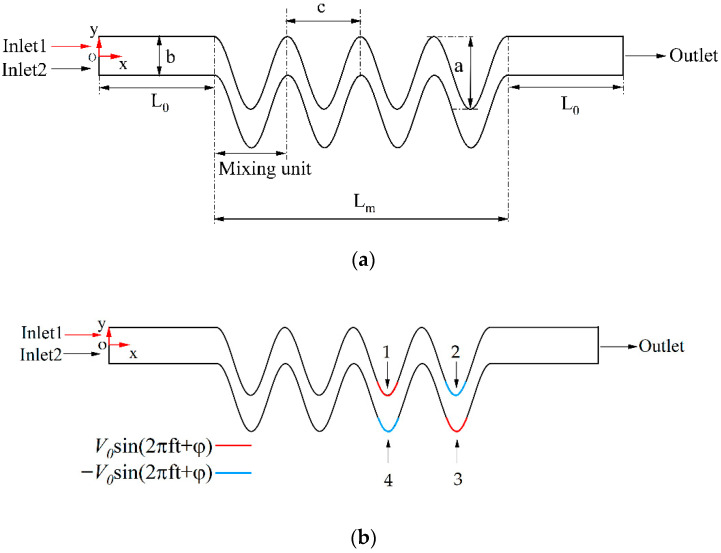
The schematic diagram of the proposed micromixers: (**a**) the passive micromixer design, (**b**) the distribution of electrodes in the active electroosmotic micromixer.

As shown in Figure 1, the schematic diagrams of a micromixer with a curved channel were proposed. To enhance the mixing performance of the micromixer, according to the convergent-divergent channel recently studied by Afzal et al. [12], the profiles of the channel walls were depicted by cosine function based on:y=acos(x);x=s×e; c=2pi×e where: y, x are the longitudinal and axial coordinates of the function, and s is defined as the axial coordinates within 0 to 2 pi. Furthermore, the values of a and c are wavy amplitude and wavelength, respectively, for all models presented in Table 1, e is defined as the distance in one wavelength, and the value is set as 50 μm. For most two-dimensional (2D) micromixers, T-shape inlets are merged in the main microchannel. In this model, as shown in Figure 1a, the two inlets, inlet 1 and inlet 2, are joined to a straight channel with L length and connected with a cosine channel. The width b of the inlet channel and mixing unit N were defined here. The length of the exit channel was the same as the inlet channel with a constant L of 500 μm. Figure 1b presents the geometric structure of the proposed active micromixer. There are two pairs of electrodes at the bottom of the cosine channel wall. The longitudinal length of the applied electrodes was 72.42 μm with reference to the bottom of the microchannel. The red electrodes 1 and 3 show positive polarity, and the blue electrodes 2 and 4 show negative voltage polarity. The alternating electric field is loaded to generate chaotic flow to promote fluid mixing.

## 3. Numerical Simulation Methods

### 3.1. Analysis Methods

To study the transport phenomenon and flow behavior of the micromixer, the Computational Fluids Dynamics (CFD) method was adopted based on the Finite Element Method (FEM). The fluid flow in the proposed micromixer was analyzed by solving the governing equations with the commercial COMSOL Multiphysics (version 5.6) software. The COMSOL Multiphysics package is capable of coupling multiple physical modules to effectively carry out numerical simulations. Besides, in this research, steady-state modeling and electric field modeling for transient states have both been considered. To reduce the considerable computational cost based on a time-dependent method and save memory and storage, two solutions were set up to investigate the mixing efficiency. A stationary solution was calculated as t = 0 s without an electric field. For the second solution, the result of the first simulation was selected as the initial value to perform the time-dependent solution together with an electric field.

### 3.2. Steady State Modeling

The first mixing process was assumed to be incompressible, viscous, and laminar in the case of a steady state. It was assumed that aqueous solutions were used here. The flow in the microchannel is governed by the Navier-Stokes equation and the continuity equation:

(1) $$\rho \vec{u}\times \nabla \vec{u}+\nabla \times \left[pI-\mu \left(\nabla \vec{u}+{\left(\nabla \vec{u}\right)}^{T}\right)\right]=0$$

(2) $$\nabla \cdot \vec{u}=0$$

With the velocity field obtained in (1), the convection-diffusion equation under steady-state flow was solved. The specific equations are as follows [26]:

(3) $$\vec{J_{i}}=-D_{i}\nabla \vec{C_{i}}$$

(4) $$\frac{\partial \vec{C}}{\partial t}=-\nabla \vec{J_{i}}-(\vec{u}\cdot \nabla )\vec{C_{i}}+R_{i}$$

where $\vec{J_{i}}$ denotes the mass flux of the $i_{th}$ species, $\vec{u}$ is the velocity of the fluid, *ρ* is the density, *µ* is the dynamic viscosity, *p* is the pressure, *I* denotes the identity tensor, and *D_i_* is the diffusion coefficient. In this case, for the steady flow state with the above convection-diffusion equation, two fluids with the equal viscosity *µ* = 0.001 Pa·s and the equal density *ρ* = 10^3^ Kg/m^3^ were used. The diffusion coefficient is taken as 10^−11^ m^2^/s. It is considered that there is no action that has an influence on the species concentration. Therefore, *R_i_* = 0 here, and the physical parameters are based on liquid water at 25 °C.

**Figure 2:**
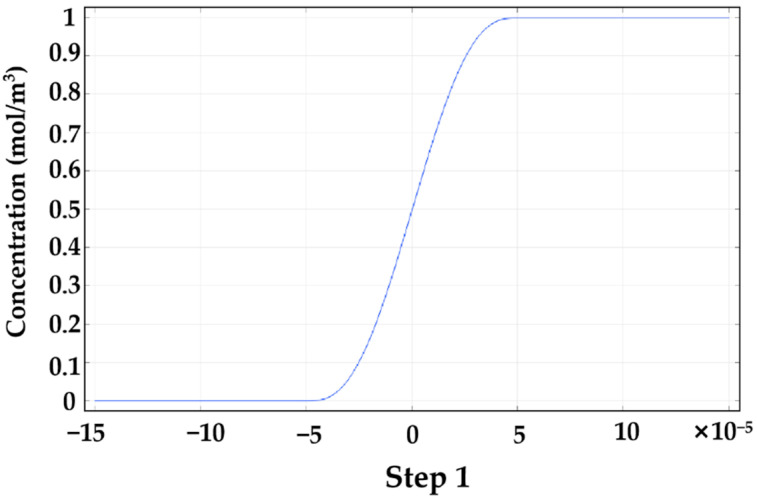
The step function of the concentration distribution on the microchannel inlet.

The step function was used to calculate the inlet concentration distribution, as shown in Figure 2. The concentration condition on inlet 1 and inlet 2 gives a sharp but smooth concentration gradient in the middle of the channel entrance. For the steady state, the molar concentration at inlet 1 and inlet 2 are set as follows:

(5) $$C_{1}=C_{0}=1\;{\mathrm{mol}/m}^{3}$$

(6) $$C_{2}=0$$

In our simulation, the identical fluid velocity of inlet 1 and inlet 2 was selected as U_0_ = U_inlet1_ = U_inlet2_ = 0.1 mm/s. The constant flow velocity and zero fixed pressure boundary condition at the outlet were specified. The no-slip boundary condition was assigned at the microchannel walls.

### 3.3. Electric Field Modeling

With a time-dependent AC electric field applied to the microchannels, the electroosmotic flow in the fluid area was driven by Multiphysics models coupling a flow field, a concentration field, and an electric field. Three Multiphysics fields correspond to the laminar flow (SPF) module, transport of diluted species (TDS) module, and electric currents (EC) module, respectively, in the COMSOL software.

The fluid flow behavior in the microchannels was governed by the Navier-Stokes equation and the continuity equation, as presented below:

(7) $$\rho \frac{\partial \vec{u}}{\partial t}+\rho \vec{u}\cdot \nabla \vec{u}+\nabla \cdot \left[pI-\mu \left(\nabla \vec{u}+{\left(\nabla \vec{u}\right)}^{T}\right)\right]=F$$

(8) $$\nabla \cdot \vec{u}=0$$

where *ρ*, *µ*, *p* and *I* are the same meaning as the steady state. In this model, the outlet pressure boundary condition sets equal to zero. In addition, *F* is the electroosmotic body forces here. However, in the passive micromixers, the surface effects are stronger than the volume effects, so it is considered that *F* = 0.

When the thickness of the EDL is much smaller than the size of the microchannel, the velocity gradient inside the EDL can be ignored. The Helmholtz–Smoluchowski equation was used to describe the relationship between the magnitude of the electric field velocity and the tangential component of the electric field [26,30]:

(9) $$\vec{u}=-\frac{\varepsilon_{0}\varepsilon_{r}\zeta }{\mu }E\left(I-nn\right)$$

where $\vec{u}$ is the fluid velocity on the channel wall, and ε_0_, ε_r_, and ζ indicate the vacuum permittivity, the relative dielectric constant of the solution, and the zeta potential of the channel wall, which are equal to 8.85 × 10^−12^ F/m, 80.2 and −0.1 V, respectively.

In this mixer, the concentration field of the mixing fluid was described by the convection-diffusion equation as the same as the steady state Equations (3) and (4).

The boundary conditions of the solute concentration at inlet 1 and inlet 2 were adopted as well as Equations (5) and (6), respectively, and no species flux boundary was applied to other walls as Equation (10):

(10) $$\vec{n}\cdot (-D_{i}\nabla \vec{C_{i}}+\vec{u}{\vec{C}}_{i})=0$$

At the outlet, the solute transport process was primarily controlled by convection. Therefore, the boundary condition was applied, as given below:

(11) $$\vec{n}\cdot (D_{i}\cdot \nabla \vec{C_{i}})=0$$

The Laplace equation was used for the AC electric field, and the electric potential *V* can be obtained as follows:

(12) $$\nabla^{2}\cdot V=0$$

With Equation (12), the electric field E can be calculated from the electric potential by Equation (13):

(13) E=−∇⋅V

Boundary conditions on the electrodes (1, 3) and (2, 4) were specified as Equations (14) and (15). Other insulated boundaries were presented by Equation (16):

(14) $$V=V_{0}\sin(2\pi ft+\varphi )$$

(15) $$V=-V_{0}\sin(2\pi ft+\varphi )$$

(16) n⋅(−σ∇V)=0

### 3.4. Mixing Efficiency Evaluation 

In order to evaluate the mixing performance of proposed micromixers, the mixing efficiency index MI is defined to quantify the mixing degree of two solutions. The mixing efficiency at the channel outlet can be calculated by the following expression [31]:

(17) $$MI=1-\frac{1}{\bar{C}}\sqrt{\frac{\sum_{i=1}^{n}(C_{i}-\bar{C}{)}^{2}}{n}}$$

(18) $$\bar{C}=\frac{1}{n}\sum_{i=1}^{n}C_{i}$$

where *MI* is the mixing efficiency, $C_{i}$ is the concentration value at each node, $\bar{C}$ is the average mass fraction across the section, and n denotes the number of sampling points in the section. No mixing is defined by *MI* = 0, whereas perfect mixing is presented by *MI* = 1. Therefore, the higher the value (close to 1) is, the better the mixing quality is. To evaluate the micromixer performance, it is important to consider the characteristic dimensionless number. The Reynolds number is defined as the following equation to represent the fluid flow in this study:

(19) $$Re=\frac{\rho \vec{u}l}{\mu }=\frac{\rho \vec{u}b}{\mu }$$

where ρ, $\vec{u}$,μ, represent the density, the velocity of the fluid, and dynamic viscosity, and l denotes the characteristic width of the channel. In this study, the microchannel width b is the characteristic width. 

### 3.5. Mesh Independency Test 

**Figure 3:**
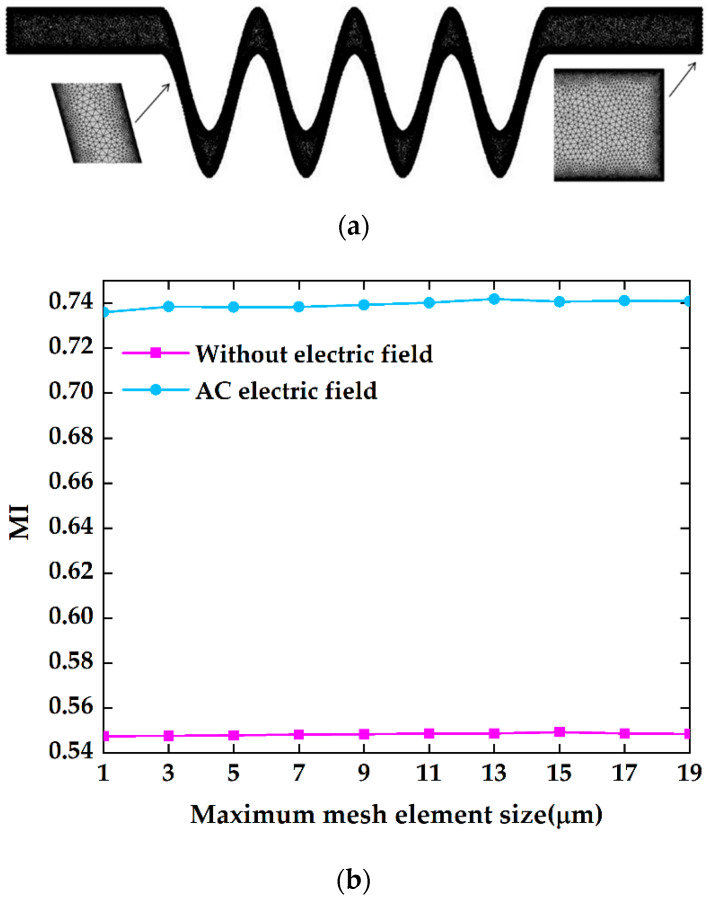
Grid system profiles: (**a**) unstructured triangular mesh, (**b**) comparison of the mixing efficiency for different grid systems.

A high-quality mesh is significant for obtaining accurate solutions. For this model, an unstructured triangular mesh was used to discretize the computational domain. In order to eliminate the influence of mesh size and quality on the modeling results, ten different structure elements experiments were carried out for different mesh resolutions for the mesh independency test. Figure 3a presents the mesh system of the customized grid result. For the purpose of gaining the high accuracy of the concentration gradient on the interface between two fluids from two inlets, the grid system was refined and modified. In this work, two grid sequences were adopted to specify the size of the mesh elements. The element size of the first grid sequence was employed for the entire geometry. The specified mesh refinements were applied to the boundary wall. Figure 3b shows the mixing efficiency of different grid systems without an AC electric field at a steady state field and applying an AC electric field, respectively. The maximum element size ranges from 1 μm to 19 μm. Compared to all mixer index results at micromixer outlets with different grid systems, the mesh element size of 9 μm can be enough to achieve reasonable accuracy. Refined mesh with 273,379 elements was employed as the optimal mesh system. Table 2 indicates detailed parameters about the element size of both grid sequences. It shows that the minimum element quality and the average element quality can be as high as 0.4588 and 0.8462, respectively, by statistics.

## 4. Results and Discussions

### 4.1. Mixing Effect of the Structures

#### 4.1.1. Mixing Effect of a/c 

**Figure 4:**
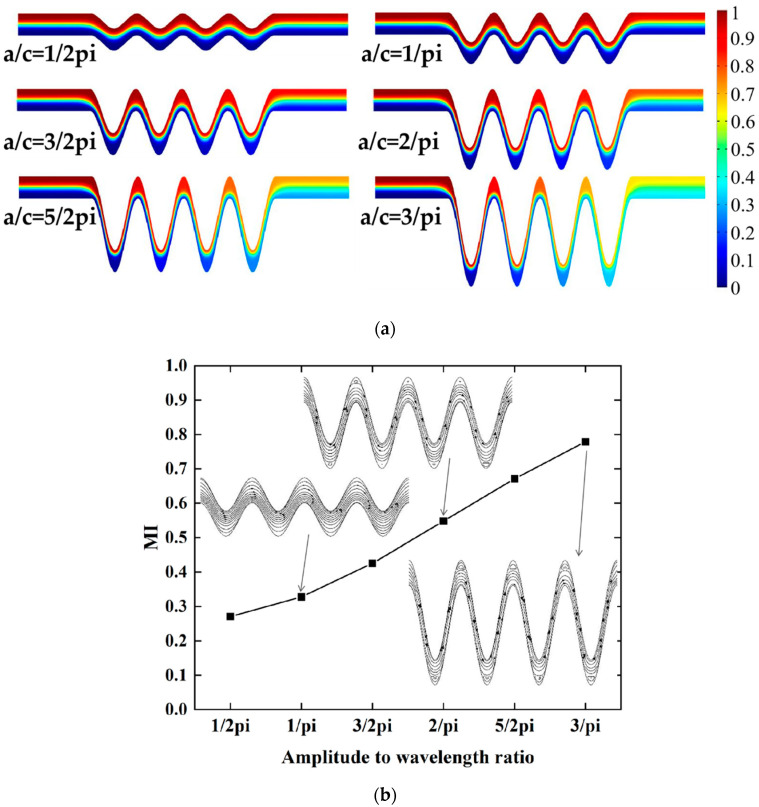
The effect of the amplitude-to-wavelength ratio: (**a**) distributions of concentration surface along the micromixer for the different amplitude-to-wavelength ratios (a/c), (**b**) the mixing index at the outlet for different values of amplitude-to-wavelength ratios (a/c).

The proposed micromixer structures are varied at different amplitude-to-wavelength ratios (a/c). The microchannel width is constant at 150 μm with mixing units of four. In this study, the micromixer amplitude-to-wavelength ratio (a/c) was one of the key parameters for structure optimization, which was increased from 1/2pi to 3/pi in Figure 4. 

Figure 4a shows the concentration distribution along the x direction in all ratios of a/c under a steady state. It can be seen from Figure 4a that the fluid flow in the micromixer is completely laminar, and the fluid mixing behavior entirely depends on intermolecular diffusion. As can be seen from Figure 4b, the mixing efficiency improved qualitatively as the ratio of amplitude-to-wavelength increased. With a/c in the range of 1/2pi to 2/pi, the two fluid flow paths were almost parallel to each other, and there was no secondary flow. When a/c = 5/2pi and 3/pi, the mixing length was elongated. Two different fluids can be relatively fully mixed, and the mixing efficiency was significantly improved. Figure 4b shows the variation of the mixing index for the six a/c mixer configurations. The mixing efficiency is proportional to the a/c ratio. When a/c is 3/pi, that is, when the amplitude is 300 μm, mixing efficiency can be achieved at 78%. The concentration streamlines were added to the mixing index curve in Figure 4b, corresponding to a/c of 1/pi,2/pi, and 3/pi. With a low amplitude-to-wavelength ratio, fluid flow was close to the upper and lower cosinusoidal microchannel walls, and it was not enough to meet the requirements to generate a vortex. The fluid stay time increased with the ratio increase, and the centrifugal force near the top and bottom was larger than that at the center of the microchannel [14,32]. The fluid was thrown to the next mixing unit, and the mixing index became larger.

#### 4.1.2. Effect of Mixing Units 

**Figure 5:**
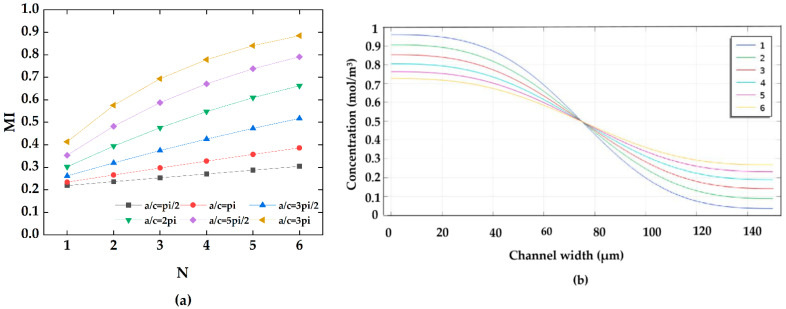
Variations of the mixing quality at the outlet with different mixing units: (**a**) the mixing index of different amplitude to wavelength ratios, (**b**) the mixing concentration at the outlet.

In passive micromixers with a steady state, laminar diffusion is the main flow mixing behavior. To a certain extent, the number of mixing units affects whether the mixer can achieve a reasonable mixing quality. In this study, the mixing performance of different mixing units was deeply studied in Figure 5. Figure 5a shows the variations of mixing efficiency at the outlet of microchannels for six mixing units for six values of a/c ratios at an inlet velocity of 0.1 mm/s. It can be clearly seen that for the proposed micromixer at any structural design, the mixing case when the mixing unit of N = 6 outperformed the other cases. In general, it can be concluded that with the increase of mixing units, the mixing quality qualitatively improves. Figure 5b presents the mixing concentration at the outlet for different mixing units. In the case of this simple micromixer, fluid mixing was not restricted at low inlet velocity by increasing the mixing path. On the other hand, the curvilinear forms of the micromixer channel increase the two fluids’ contact area and thus enhances the mixing performance. As can be seen from Figure 5b, a more sufficient mixing effect can be achieved with the mixing units of 6.

### 4.2. Electroosmotic Flow Mixing 

#### 4.2.1. Model Validation

**Figure 6:**
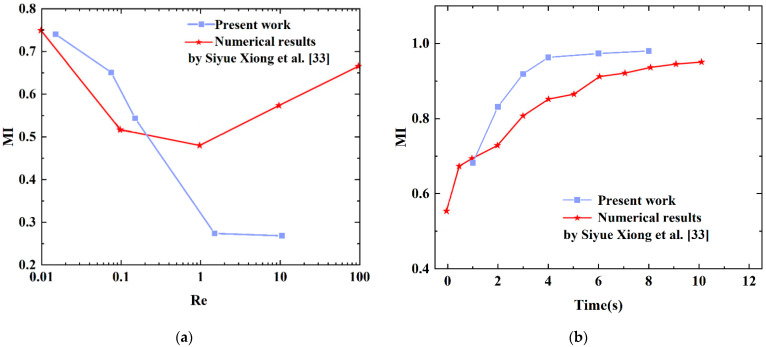
Model validation based on Siyue Xiong et al. [33] (**a**) comparison of the mixing index for different Re numbers, (**b**) comparison of the mixing index for different voltages.

A validation for electroosmotic flow mixing is presented by comparing the electroosmotic micromixer simulated by Siyue Xiong et al. [33]. As is seen in Figure 6, the effect of different values of Reynolds number from 0.015 to 10.5 and voltage from 1 V to 8 V is shown through quantitative comparison. The mixing effect of the micromixer is closely related to the variations of the Reynolds number. At low Reynolds numbers from 0.01 to 0.15, present results are consistent with the simulation results in [33]. With the increase of Reynolds number at a low range, the mixing index decreases. Similarly, in Figure 6b, the value of the voltage is proportional to the mixing efficiency. This model illustrates that the present work agrees with those investigated by Siyue Xiong et al. [33].

**Figure 7:**
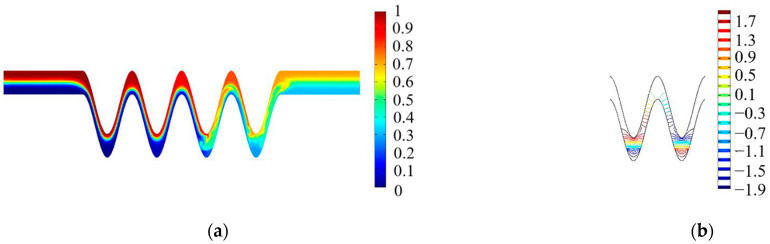
Simulation of the electroosmotic micromixer: (**a**) distribution of concentration surface along the micromixer, (**b**) electric potential streamlines when time t = 1 s, phase shift of pi/4, U_0_ = 0.1 mm/s, *V*_0_ = 2 V, f = 5 Hz.

**Figure 8:**
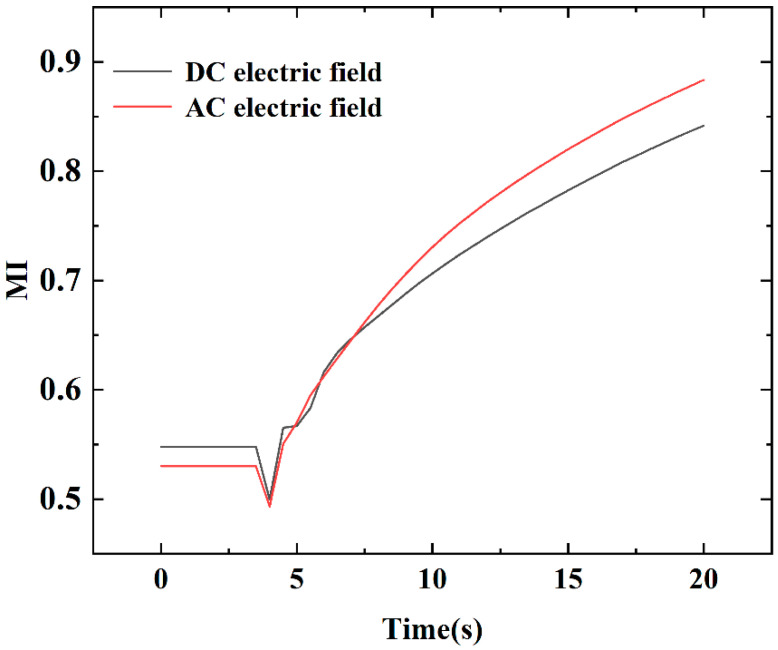
Comparison of the mixing efficiency at the outlet of the proposed micromixer under an AC electric field and a DC electric field with U_0_ = 0.1 mm/s, *V*_0_ = 4 V, f = 5 Hz.

In this study, it can be seen from the above research that adjusting the main parameters of the micromixer structure, including the amplitude-to-wavelength ratio a/c and the mixing unit N, can play a role in obtaining a high mixing index of the proposed micromixer. However, under the appropriate microchannel structure parameters, such as the ratio of a/c of 2/pi and the value of b at 150 μm, the blending effect can only be at a medium level. Moreover, in the steady state, when the number of mixing units is at six, a better mixing effect can be achieved in all cases. Assuming that external energy is added to become an electroosmotic flow active micromixer, the mixing index will have a chance to improve further under the above cases. To shorten the mixing channel length while increasing the mixing efficiency, the number of mixing units is kept at four. The flow field, concentration field, and electric field of the electroosmotic micromixer can be calculated according to the governing equations given in (Section 3.3). In this part of the simulation, as shown in Figure 2, the values of amplitude-to-wavelength ratio (a/c) and channel width (b) were 2/pi and 150 μm, respectively, which were fixed at appropriate values. Concentration profiles in Figure 7a present that when an AC electric field is applied, the vortices are generated primarily in the environs of the electrodes, which develop into waves within the side wall of the cosine microchannel and continue to the straight microchannel. As shown in Figure 7b, a relatively uniform electric intensity is formed. To assess the mixing efficiency of the present electroosmotic micromixer, a comparison of time-dependent variations of both AC and DC electric fields was presented in Figure 8. As can be seen from Figure 8, AC electroosmosis has a better induction effect than DC electroosmosis.

#### 4.2.2. Mixing Effect of Reynolds Number 

**Figure 9:**
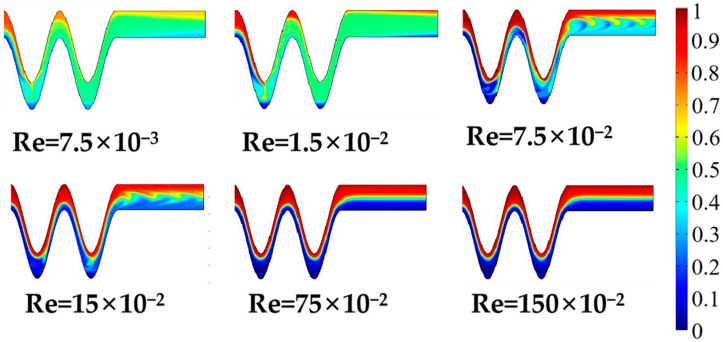
Distributions of concentration surface from the A-A section to the outlet of the micromixer for different inlet velocities when time t = 10 s, phase shift of pi/4, *V_0_* = 2 V, and f = 5 Hz.

**Figure 10:**
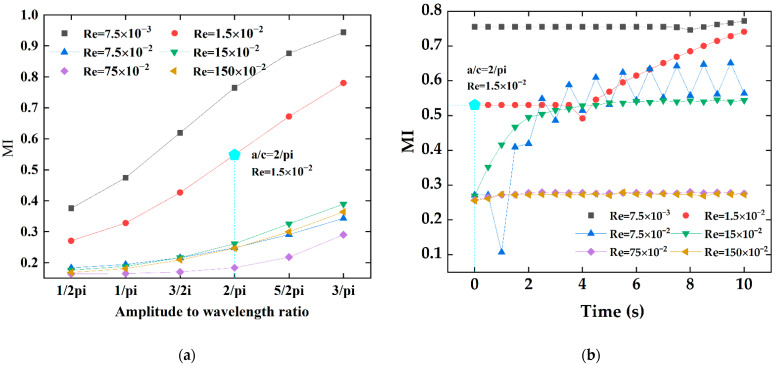
Variations of the mixing index at the outlet of the passive micromixer and the electroosmotic micromixer with different Reynolds numbers: (**a**) the mixing index of the passive micromixer of six amplitude of wavelength ratios, (**b**) the mixing index of the electroosmotic micromixer within 0–10 s.

In the present study, in order to further describe fluid flow characteristics within the micromixer, the Reynolds number should be further studied to understand the fluid flow phenomenon. Figure 9 presents concentration distribution profiles from the A-A section to the outlet of the electroosmotic micromixer under six different Reynolds numbers ranging from 7.5 × 10^−3^ to 150 × 10^−2^. The Re number of 1.5 × 10^−2^ corresponds to the inlet velocity of 0.1 mm/s. The fluid mixing becomes more uniform related to Reynolds numbers of 7.5 × 10^−3^, 1.5 × 10^−2^ correspondingly to the inlet velocity of 0.05 mm/s and 0.1 mm/s, respectively. With Reynolds numbers increasing to 7.5 × 10^−2^ and 15 × 10^−2^, the fluid distribution is plug-like and continues to the channel outlet. When the Reynolds number is larger than 15 × 10^−2^, the fluid concentration basically does not change and shows basic lateral flow. It can be seen from the results that the mixing quality at the outlet of the micromixer decreases as the Re numbers increase. This is due to the dominance of electroosmotic force at low velocities, and the mixing time is extended, leading to increased mixing quality. However, as the Reynolds number increases, the eddy generated by the electroosmotic force cannot effectively induce fluid mixing, and the laminar flow plays a major role at this time. As seen in Figure 10, the mixing efficiency of the proposed micromixers with steady and transient states was compared. In order to further investigate the influence of the Reynolds inlet on the mixing performance, numerical simulations of the micromixer at different amplitude-to-wavelength ratios of each case were carried out. It is easy to know that smaller inlet velocity shows a better blending effect in all cases. By comparing Figure 10a,b, the mixing index of the electroosmotic micromixer in the transient state exceeds that of the passive micromixer in the steady state. At a steady state, the increase in flow velocity makes laminar diffusion weaker, resulting in the reduction of the mixing quality. The fluid direction changes due to the alternating electric field. A Reynolds number of 7.5 × 10^−3^ and an inlet velocity of 0.05 mm/s produced a mixing efficiency of 77%, while the mixing index for a Reynolds number of 1.5 × 10^−2^ is reduced by 22% compared with 7.5 × 10^−3^. It can be concluded that the mixing quality can be effectively improved by reducing the inlet velocity.

#### 4.2.3. Mixing Effect of the Number of Electrode Pairs 

**Figure 11:**
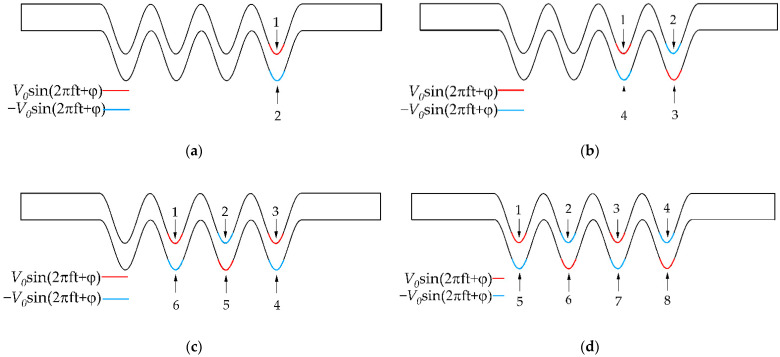
Distributions of electrode pairs positions: (**a**) one pair, (**b**) two pairs, (**c**) three pairs, (**d**) four pairs.

**Figure 12:**
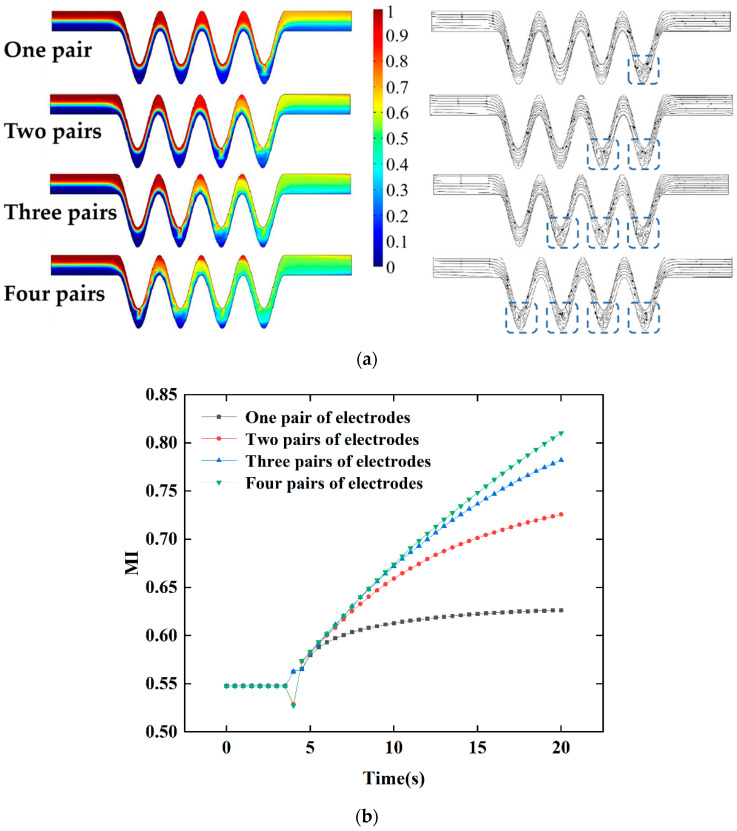
The effect of the number of electrode pairs: (**a**) distributions and streamlines of concentration surface along the micromixer for different electrode pairs, (**b**) the mixing index at the outlet for different electrode pairs within 0–20 s when phase shift of pi/4, U_0_ = 0.1 mm/s, *V*_0_ = 2 V, and f = 5 Hz.

Next, based on the previous simulation conclusions, it is supposed that applying multiple electrode pairs on the proposed micromixer has an effect on the mixing efficiency. Figure 11 shows the positions of the different electrode pairs. Previously, two pairs of electrodes were set up. Based on the physical model in Figure 2, under the same conditions, four pairs of electrodes were set up. The alternating electric field voltage amplitude value and frequency were also kept at 2 V and 5 Hz. Figure 12a shows the concentration profiles of microchannels with four electrode pairs at t = 20 s, respectively. It can be seen from Figure 12 that when the electrode pairs were applied, the fluid concentration distribution at the bottom of the channel changed significantly, while there was no obvious mixing behavior at the wall of straight channels and cosine microchannels away from the electrodes. As the number of electrode pairs increased, the fluids formed a vortex near the electrode, causing the fluids to be stretched and folded repeatedly. The fluid mixing contact area increased, and the mixing effect was considerable. The concentration streamlines for different pairs of electrodes in Figure 12a have been presented. It can be observed that the electric field had a significant impact on fluid mixing throughout the micromixer. As depicted in Figure 12b, the results show that when a pair of electrodes is applied, the mixing efficiency can reach 62% at t = 20 s, while more pairs of electrodes on the microchannel lead to a small increment of the mixing efficiency. The rotating vortex at this time weakens the mixing effect of the fluids, which is caused by the mutual influence of adjacent electrode pairs on the fluid disturbance in the microchannel. When four pairs of electrodes are applied, the mixing efficiency reaches 82%, which is a 10% increase over when two pairs of electrodes are applied.

#### 4.2.4. Mixing Effect of Phase Shift 

**Figure 13:**
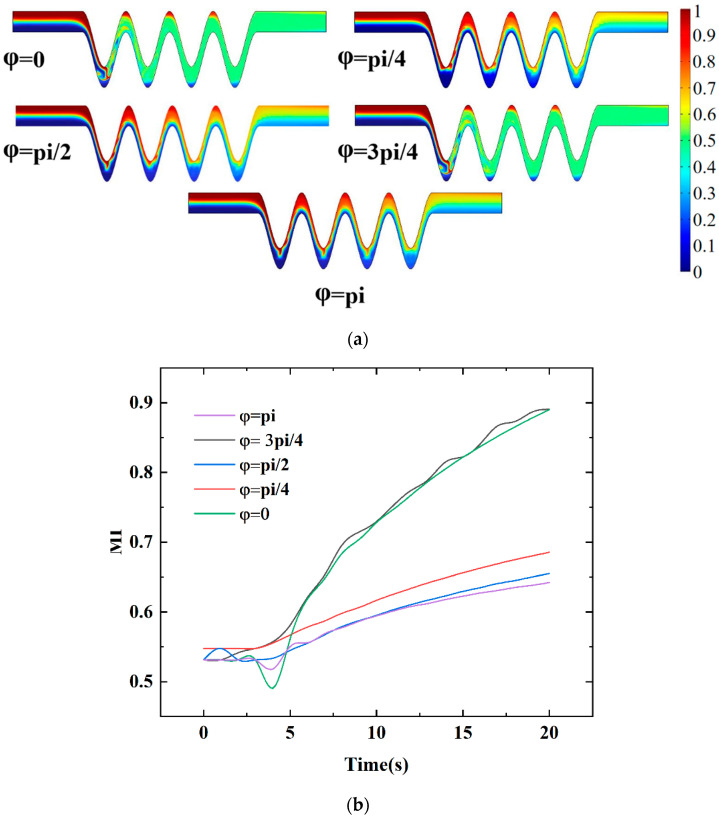
The effect of phase shift: (**a**) distributions of concentration surface along the micromixer for different phase shifts, (**b**) the mixing index at the outlet for different phase shifts within 0–20 s when four pairs of electrodes, U_0_ = 0.1 mm/s, *V*_0_ = 2 V, and f = 5 Hz.

Besides, the effect of phase shift on mixing efficiency has been carried out. In this part, the 1, 3, 5, and 7 boundaries of the micromixer in Figure 11d are set as ground, that is, zero potential. The 2, 4, 6, and 8 boundaries are set as periodic potentials with the opposite polarity of adjacent electrodes. Moreover, the potential function of boundary 2 is kept as the initial phase so that its phase shift with other potential functions is 0, pi/4, pi/2, 3pi/4, and pi, respectively. As shown in Figure 13a, different phase shifts were tried. Four pairs of electrodes were used. It can be seen from Figure 13 that when the phase shift of the potential function at the bottom side is changed, the mixing efficiency at the outlet changes. When the phase shift is 0 and 3pi/4, the effect of electroosmosis and external electric field on the fluid mixing is relatively stronger. An obvious mixing efficiency larger than 90% can be achieved. When the phase shift of pi/4, pi/2, and pi, the internal disturbance of the fluid under the action of the vortex was strengthened so that the mixing effect was enhanced. When the phase shift is pi/4, the size of the fluid vortex increases starting from the first mixing cycle unit, and the highly concentrated fluid layer stretches outward for effective mixing. Figure 13b shows the mixing efficiency within t = 20 s for different phase shifts. As expected, in the range of 0 to pi phase shift, the electroosmotic and external electric field driving forces generated by varying the phase shifts are proportional to the mixing index. The phase shift of 3pi/4 obtains the best mixing for a short time with respect to a mixing index of 90.3%. It can be seen from Figure 13b that the mixing can be effectively induced by adjusting the phase shift.

#### 4.2.5. Mixing Effect of the Voltage

**Figure 14:**
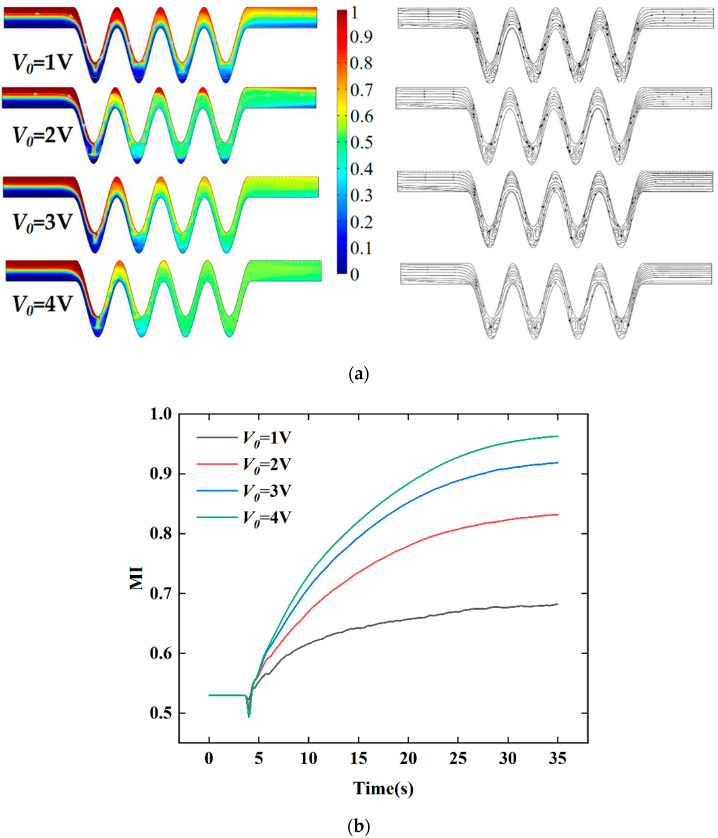
The effect of the voltage: (**a**) distributions of concentration surface along the micromixer for four values of voltage, (**b**) the mixing index at the outlet for different voltages within 0–35 s with four pairs of electrodes, and a phase shift of pi/4, U_0_ = 0.1 mm/s, *V*_0_ = 2 V, and f = 5 Hz.

Next, in order to further study the effect of applying electroosmotic flow and AC electric field on the micromixer, different voltages in the range of 1–4 V were investigated to find its motion of the effect on the fluid mixing behavior. In this part, no phase shift was set to explore the effect of voltage on mixing properties. Figure 14a,b present the behavior of the fluid flow in the electroosmotic micromixer in eight cases. The frequency and the mean velocity were kept at 5 Hz and 0.1 mm/s, respectively. As shown in Figure 14a, the mixing index was developed with increased electric voltages. When the voltage was high, the vortex transported the highly concentrated fluid backward, blurring the boundaries between the fluids and getting well mixed. The vortex produced by the electroosmotic flow squeezed the high-concentration fluid downward, significantly improving the mixing quality. The streamline diagrams on the right of Figure 14a show that several pairs of symmetrical vortices were formed near the electrodes at the bottom of the microchannel, and the fluid streamlines were stretched in opposite directions. Moreover, the influence of the fluid vortex gradually expanded as the voltage increased, presenting a fold and stretch motion. The mixing efficiency for different voltages applied on the electroosmotic micromixer is quantitatively compared. When the voltage of *V*_0_ = 2 V, the mixing quality can reach 83%. When beyond the value of the electric voltage of *V*_0_ = 3 V, the mixing degree at the outlet of the micromixer can be greatly mixed. Subsequently, the mixing efficiency gradually tends to be stable.

#### 4.2.6. Mixing Effect of the Frequency 

**Figure 15:**
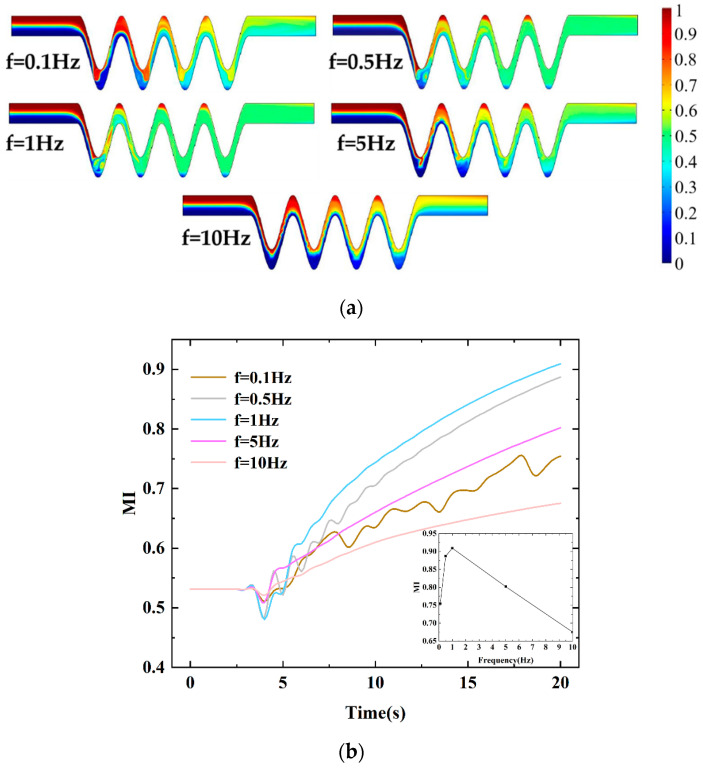
The effect of the frequency:(**a**) distributions of concentration surface along the micromixer for different frequencies, (**b**) the mixing index at the outlet for different frequencies within 0-20 s with four pairs of electrodes and a phase shift of pi/4, U_0_ = 0.1 mm/s, and *V*_0_ = 2 V.

In addition to the above factors affecting mixing efficiency, different AC frequencies were also investigated from 0.1 Hz to 10 Hz to study which frequency would achieve suitable mixing quality. Figure 15 shows the concentration distribution of the proposed micromixer with five frequencies to study the effect of frequencies on the mixing quality. The results show that the mixing index increased when the frequency increased from 0.1 Hz to 0.5 Hz and then decreased when the value was from 1 Hz to 10 Hz. It is worth noting that when the frequency increased, the mixing efficiency did not increase as we would expect. It can be seen from Figure 15a that the concentrations can be uniformly distributed, which indicates that the fluids achieve good mixing. When the frequency was increased to f = 5 Hz and f = 10 Hz, the mixing effect was not ideal. When the applied frequency was 10 Hz, the two fluids rotated near the electrodes. From Figure 15b, it can be demonstrated that an optimal mixing index of 91% is obtained when f = 1 Hz. Figure 15b also extracts the mixing efficiency at the outlet when the mixture reaches a relatively stable state. Generally, at low frequencies, there is an optimal frequency contributing to the maximum mixing efficiency. This is because there is insufficient time to respond and make the electroosmotic flow create rotating vortices under high frequency, corresponding to a smaller period [29,34,35]. This may contribute to the change of positive and negative polarity of electrodes when the electric field changes faster with the increase of the frequency.

## 5. Conclusions

This paper proposed micromixers with cosine microchannel walls and conducted various simulations based on a steady state without an electric field and a transient state with an AC electric field to investigate the mixing characteristics. From the results of the above research about structural designs of the micromixer in a steady state, adjusting optimal parameters can effectively improve the mixing performance. The results show that a high amplitude-to-wavelength ratio and more mixing units result in effective mixing. It is worth noting that no secondary flow and vortices are generated in the mixer without an electric field driving force. To further obtain a higher mixing index, electrode pairs were applied to the walls of the microchannel of the proposed micromixer, which became an electroosmotic micromixer. The micromixer designs for the above parameters were studied based on a/c of 2/pi, b of 150 μm, and N of 4. It was demonstrated that the vortices created by the application of an AC electric field would greatly facilitate mixing. The influence of a Reynolds number, the number of electrodes, electric phase shift, frequency, and electric voltage were performed. The simulation results show that for a Reynolds number larger than1.5 × 10^−2^ in the range of 7.5 × 10^−3^ to 150 × 10^−2^, the molecular diffusion did not result in high efficiency. Moreover, more pairs of electrodes can reveal a better effect. Also, the mixing was enhanced significantly with the phase shift of 3pi/4 combined with four electrodes yielding more electroosmotic vortices. Surprisingly, the results also indicate that as the relatively low-frequency values decrease with the range of 0.1 Hz to 10 Hz, the mixing performance first performs better and then becomes poor. A 1 Hz frequency can achieve a mixing index of 91%. Based on the research on voltages, the mixing effect is in proportion to the voltages. The voltage of 4 V with the range of 1 V to 4 V leads to a maximum mixing efficiency of greater than 96%. This electroosmotic micromixer presents a simple structure with remarkable mixing characteristics. Further investigations about the structure and parameters optimization would be studied to enhance the mixing quality.

## Figures and Tables

**Table 1:** Dimensions for the proposed micromixers.

Dimensions	Value	Unit
Wavelength (c)	2pi×e	μm
Amplitude (a) to a/c ratio of 1/2pi	50	μm
Amplitude (a) to a/c ratio of 1/pi	100	μm
Amplitude (a) to a/c ratio of 3/2pi	150	μm
Amplitude (a) to a/c ratio of 2/pi	200	μm
Amplitude (a) to a/c ratio of 5/2pi	250	μm
Amplitude (a) to a/c ratio of 3/pi	300	μm

**Table 2:** Detailed parameters of element size in the grid system.

Element Size	Maximum Element Size (μm)	Maximum Element Growth Rate
Whole geometry	9	1.3
Microchannel boundaries	0.5	1.1

## Data Availability

Not applicable.
